# Supplementary material for: The Sec14-like phosphatidylinositol transfer proteins Sec14l3/SEC14L2 act as GTPase proteins to mediate Wnt/Ca2+ signaling
Source: eLife. 2017 May 2;6:e26362. doi: 10.7554/eLife.26362 (PMC5423769; doi:10.7554/eLife.26362)
Supplement: Supplementary file 1. — DOI: http://dx.doi.org/10.7554/eLife.26362.025 [file elife-26362-supp1.docx]

**Supplementary file 1:**

**Primers used in the present study:**

| Construct name |  | Primer sequences |
| --- | --- | --- |
| pCS2-Flag-Sec14l3-ΔS | F | CCGGAATTCATGCTGACCGATCCTGATGGGG |
|  | R | CCGCTCGAGCTAGTTGTCTGATTGGTTGAC |
| pCS2-Flag-Sec14l3-ΔG | F | CCGGAATTCATGAGCGGAAGGGTTGGAGATC |
|  | R | CCGCTCGAGCTAACAGTTTGGTGCGATAAGCTC |
| pCS2-Flag-Sec14l3-ΔGα | F | CTAGGATTAAAGTGGACTATGAACAGTCTG |
|  | R | ATAGTCCACTTTAATCCTAGTTCTACATTTGG |
| pCS2-Flag-Sec14l3-ΔNG | F | CCGGAATTCATGATAGAGTTTCGGAAACACATG |
|  | R | CCGCTCGAGCTAACAGTTTGGTGCGATAAGCTC |
| pCS2-mDvl2-Myc | F | CGGAATTCGCGGGCAGCAGCGCGGGGGG |
|  | R | CGCGGATCCAAAAAATACTCCCCCAAATAT |
| pCS2-Plcδ4a-N2-HA | F | CCGGAATTCATGACAGAACGACAGATGGCTTC |
|  | R | CGCGGATCCGCGCTGAGTCAGCATCTTA |
| pCS2-Plcδ4a-N1-HA | F | CCGGAATTCATGACAGAACGACAGATGGCTTC |
|  | R | CGCGGATCCGAGTTCGAGCATTGCAGGGTTG |
| pCS2-Plcδ4a-C1-HA | F | CCGGAATTCATGTACCAAGACATGTCTCAGCCTC |
|  | R | CGCGGATCCGGTTAAGTTTGTAATCCGGATATG |
| EZ-T-Sec14l3 | F | ATAGAGACTTATGGAGAGGTTC |
|  | R | CTGATTGGTTGACTTGACTG |
| pXT7-Plcδ4a-Δ28-HA | F | GCGATGATTTAATCCACCTATCTTCTTTC |
|  | R | TAGGTGGATTAAATCATCGCAACAACAGC |
| pXT7-PLCδ1a-PH-mCherry | F | CGGAATTCCACCATGGACTCGGGCCGGGAC |
|  | R | GAAGATCTTTACTTGTACAGCTCGTCC |
| pXT7-GCaMP6 | F | GAAGATCTCACCATGGGTTCTCATCATCATC |
|  | R | GGACTAGTTCACTTCGCTGTCATCATTTG |
| pXT7-AKT1-PH-mCherry | F | CGGAATTCCACCATGAGCGACGTGGCTATT |
|  | R | GAAGATCTTTACTTGTACAGCTCGTCC |
| pXT7-Sec14l3-ΔG-flag | F | CCGGAATTCCACCATGAGCGGAAGGGTTGGAGATC |
|  | R | CCGCTCGAGCTATTTGTCATCGTCGTCCTTGTAGTCCATACAGTTTGGTGCGATAAG |
| pEGFP-N3-Sec14l3-5' UTR | F | CGAATTCTGCAGTCGACGGTAATTTCTGCTGCACTCGTGGC |
|  | R | CTCCTCGCCCTTGCTCACCAT CTGCCACTCGGTTGTGATGG |
| pGEX-6p-1-6xMyc-hFz5-CT | F | GGAATTCTCGGGCAAGACGGTGGAG |
|  | R | CCGCTCGAGCTACACGTGCGACAGGGACAC |
| pGEX-6p-1-6xMyc-RFz2-CT | F | GGAATTCTCCGGCAAGACGCTGCAC |
|  | R | CCGCTCGAGTCACACGGTGGTCTCTCC |
| pCS2-Plcδ1a-HA | F | CCATCGATATGTCTTGTCCATTTAAACCTC |
|  | R | CCGCTCGAGAGCATCTAGAAGCATAACGTG |
| pCS2-Plcδ3b-HA | F | CCATCGATATGTTGAGAAAGAAGAAGACTG |
|  | R | CCGCTCGAGTTCTCGGGCACTATGTGCGAC |
| pCS2-Plcδ4a-HA | F | CCGGAATTCATGACAGAACGACAGATGGCTTC |
|  | R | CGCGGATCCGGTTAAGTTTGTAATCCGGATATG |
| pCS2-Flag-Sec14l3-ΔN | F | CCGGAATTCATGATAGAGTTTCGGAAACACATG |
|  | R | CCGCTCGAGCTAGTTGTCTGATTGGTTGAC |
